# Immunologic and Virologic Progression in HIV Controllers: The Role of Viral “Blips” and Immune Activation in the ANRS CO21 CODEX Study

**DOI:** 10.1371/journal.pone.0131922

**Published:** 2015-07-06

**Authors:** Nicolas Noel, Nathalie Lerolle, Camille Lécuroux, Cécile Goujard, Alain Venet, Asier Saez-Cirion, Veronique Avettand-Fenoël, Laurence Meyer, Faroudy Boufassa, Olivier Lambotte, ANRS C021 CODEX study group

**Affiliations:** 1 UMR 1184, Immunologie des Maladies Virales et Autoimmunes (IMVA), Université Paris Sud, Le Kremlin Bicêtre, France; 2 Assistance Publique–Hôpitaux de Paris, Service de Médecine Interne, Hôpitaux Universitaires Paris Sud, Le Kremlin-Bicêtre, France; 3 Faculté de Médecine Paris Sud, Le Kremlin Bicêtre, France; 4 INSERM U1018, Centre de recherche en Epidémiologie et Santé des Populations, Université Paris Sud, Le Kremlin Bicêtre, France; 5 Institut Pasteur, Unité HIV, Inflammation et Persistance, Paris, France; 6 Université Paris Descartes, Sorbonne Paris Cité, Faculté de Médecine, EA 7327, Paris France; 7 Assistance Publique–Hôpitaux de Paris, Service de Virologie, Hôpital Necker–Enfants Malades, Paris, France; University of Pittsburgh Center for Vaccine Research, UNITED STATES

## Abstract

Some HIV controllers (HICs) experience CD4+T cell count loss and/or lose their ability to control HIV. In this study, we investigated the rate of immunologic and/or virologic progression (ImmP/VirP) and its determinants in the ANRS CO21/CODEX cohort. Immunologic progression was defined as a lasting fall in CD4+T cell count below 350/mm^3^ or more than 200/mm^3^ with a baseline count below 600/mm^3^. Virologic progression was defined as a HIV viral load (VL) above 2000 copies/mL on two consecutive determinations. Clinical characteristics, immune activation, ultrasensitive HIV VL and total HIV DNA were analyzed. Disease progression was observed in 15 of the 217 patients followed up between 2009 and 2013 (ImmP, n = 10; VirP, n = 5). Progressors had higher ultrasensitive HIV RNA levels at inclusion (i.e. 1-2 years before progression) than non-progressors. ImmP had also lower CD4+T cell nadir and CD4+T cell count at inclusion, and VirP had higher HIV DNA levels in blood. T cell activation and IP10 levels at inclusion were significantly higher in ImmP than in non-progressors. In summary, the lasting loss of CD4+T cells, residual HIV replication and basal levels of immune activation appear to be major determinants of progression in HICs. These factors should be considered for adjusting their follow-up.

## Introduction

Most untreated HIV individuals experience continuous viral replication and thus progressive CD4+ T cell depletion. The rare HIV controllers (HICs) display spontaneous, long-term control of viremia (below 400 HIV RNA copies/mL in the French cohort) [1–3] despite being usually infected with replication-competent viruses [4]. Most HICs display potent antiviral immune responses, which are mediated by high-avidity, polyfunctional cytotoxic CD8+ and CD4+ T cells [5–9]. The patient's genetic background also influences viral control by HICs, since the HLA-B57 and B27 alleles are over-represented in these individuals [2,10].

However, some HICs eventually exhibit a decline in their CD4+ T cell count during the period of HIV control and/or lose the ability to control HIV [11,12]. Using ultrasensitive techniques, low levels of HIV replication can be detected in HICs and might be correlated with the CD4+ T cell loss [11,13]. Chronic immune activation is also observed in HICs and may contribute to both CD4+ T cell loss [14,15] and cardiovascular events [16,17].

Relatively little is known about the long-term prognosis of HICs. In a collaborative study of large cohorts of seroconverters, 25% of HICs had lost their “controller status” 20 years after seroconversion [18]. There are a few reports of disease progression in some HICs [14,19–21]. Immunologic progression (i.e. a decline in the CD4+ T cell count) despite low or undetectable viral loads (VLs) and/or the loss of control of HIV replication may occur. These situations may be transient and correspond to a “blip” of viral replication [11,18,21]. Progression can be favored by a concomitant disease, such as hepatitis C [22,23], HIV superinfection [24,25], or the initiation of particular medications (e.g. immunosuppressive drugs following organ transplantation) [26]. However, there are few data on the risk factors for immunologic and/or virologic progression in HICs. Understanding the mechanisms leading to disease progression in HICs could help to (i) identify the major determinants of HIV control and (ii) optimize care for these uncommon patients.

To address these issues, we investigated the rate of immunologic and/or virologic progression and its determinants in the French CODEX cohort of HICs (the ANRS CO21 study). We focused on (i) clinical and biological events preceding the progression, and (ii) the ultrasensitive HIV VL, HIV DNA levels, T cell activation and plasma levels of inflammatory biomarkers at enrollment. The changes over time in these parameters after immunologic/virologic progression in these patients are also described.

## Patients and Methods

### The ANRS CO21 CODEX cohort

In this study, we analyzed the 217 first HICs enrolled in the French multicenter ANRS HIV Controller CO18/CO21 CODEX cohort, which was initiated in 2009. To be enrolled, patients had to be combined antiretroviral therapy (cART)-naïve, with a diagnosis at least 5 years before enrollment and a VL below 400 copies/mL in the five preceding consecutive measurements. Each participant provided a written consent to participate in the study. The study's objectives and procedures were approved by the local investigational review board (*Comité de Protection des Personnes Ile-de-France VII*, Paris, France; reference 05–22) and the study itself was performed in compliance with the tenets of the Declaration of Helsinki.

### Immunologic/virologic progression

After enrollment in the CODEX cohort, suspected immunologic progression was defined as a single CD4+ T cell count below 350/mm^3^ or a decline of more than 200/mm^3^ from an immediately preceding CD4+ count of at least 600/mm^3^. Suspected viral progression was defined as one HIV RNA measurement above 2000 copies/mL. If both criteria were met, patients were considered to experience a suspected combined progression.

Such cases were referred to as confirmed immunologic and/or virologic progression if the above criteria were met for two consecutive blood samples. If cART had been initiated before the second (confirmatory) blood sample had been analyzed, the progression was classified as a suspected progression.

For the analysis of T cell activation and inflammatory biomarker levels, the control groups consisted of (i) viremic (VL>10,000 copies/mL), treatment-naive patients ("viremic patients"), and (ii) patients having received cART for at least one year and with no detectable VL (<50 copies/mL) ("ART-treated patients"), as described elsewhere [15,27].

### Data collection

Clinical characteristics (age, gender, HCV co-infection, comorbidities, and HIV history (year of HIV diagnosis, CD4+ nadir, the number and intensity of transient detectable plasma VLs (blips), and the baseline CD4+ count and HIV RNA VL) were recorded. The parameters of interest (CD4+ and CD8+ T cell counts and the HIV VL before progression, at the time of the suspected progression, at the next visit, and at the last measurement) were documented. The cART regimen and initiation date were also noted. A standardized data collection form was provided for the investigation of any clinical events occurring in the three months prior to the suspected progression (e.g. unprotected sex intercourse, intravenous drug use, concurrent infectious events, vaccinations, treatment changes, etc.). Since the start of inclusion in 2009, blood samples have been collected each year. Additional samples were collected for viral and immunologic analysis at the time of the suspected progression, again one month later (to confirm or refute the progression) and then when ART was initiated.

### Immunologic and virologic assays

CD4+ T cell counts were determined using standard flow cytometry procedures. The ultrasensitive HIV RNA VL was measured using ultrasensitive real-time PCR (Generic HIV Charge Virale, Biocentric, Bandol, France). HIV-1 RNA was extracted from a 15 mL plasma sample (after centrifugation for 90 minutes at 15,000 rpm) with the QiAamp Viral RNA Mini kit (Qiagen, Courtaboeuf, France) and tested in five PCRs with a quantification threshold of 1 copy/mL. Total cell-associated HIV-1 DNA was quantified as described elsewhere (Generic HIV DNA CELL, Biocentric, Bandol, France) [28].

The plasma levels of cytokines and chemokines were measured in a FlowCytomix bead-based multiplex immunoassay (IP10, TNFα, IL6, MCP1, IL10: eBioscience Inc., San Diego, CA, USA), or specific ELISAs (Human IL6 Platinum ELISA, eBioscience, CRP Gen.3 kit, Roche Diagnostics, Indianapolis, IN, USA; sCD14 and sCD163 Duokine ELISAs, R&D Systems, Minneapolis, MN, USA), as described elsewhere [15]. Surface expression of T lymphocyte activation markers (HLA-DR and CD38) was analyzed by flow cytometry of whole blood samples, as described elsewhere [29]. The HIV-specific CD8+ T cell response was measured by ELIspot IFN-γ assay using pools of optimal peptides (depending on the subjects’ HLA and HIV protein), as described elsewhere [27].

The *ex vivo* capacity of HIV-specific CD8+ T cells to suppress HIV-1 infection of autologous CD4+ T cells was assessed as described elsewhere [29,30].

### Statistical analyses

Data are expressed as the median [1^st^-3^rd^interquartile range (IQR)] for continuous variables, and *n* (%) for categorical variables. Intergroup comparisons of continuous and categorical data were compared using Mann-Whitney, chi-squared or Fisher tests, as appropriate. The threshold for statistical significance was set to *p*<0.05 for all analyses. Data were entered and analyzed using PRISM software (version 5, GraphPad Software, La Jolla, CA).

## Results

### Characteristics of the study population

**Fig 1:**
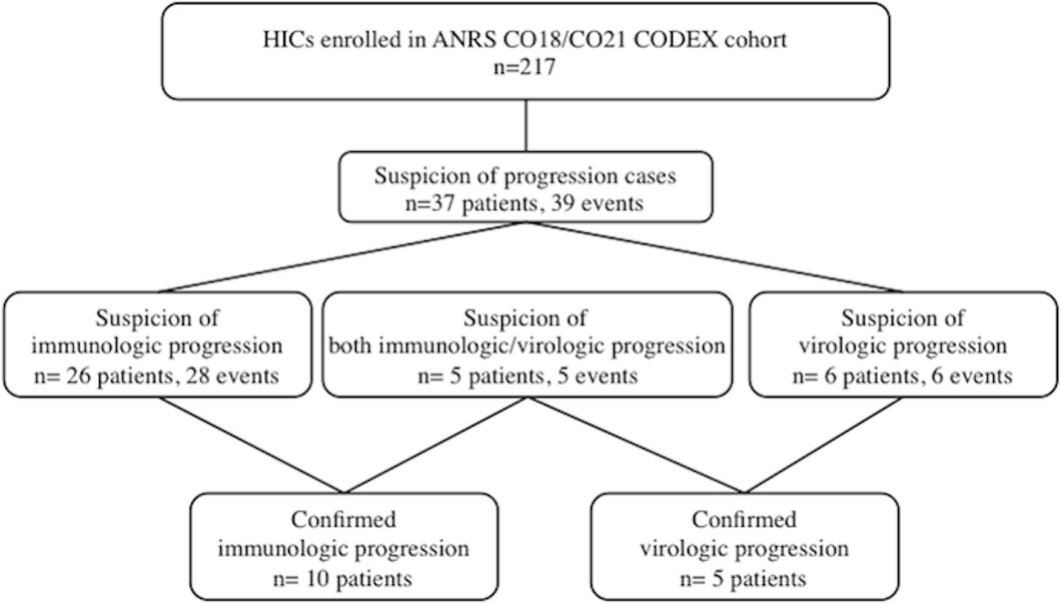
Study flow diagram. ANRS: *Agence Nationale de Recherche sur le SIDA et les Hépatites Virales*, cART: antiretroviral therapy, CODEX: *Cohorte des Extrêmes* study, HICs: HIV controllers.

We recorded all suspected progressions among the CODEX HICs until June 30^th^, 2013. As shown in Fig 1, 37 out of the 217 HICs experienced at least one suspected progression. Twenty-six patients experienced at least one or two drops in their CD4+ T cell count (this situation occurred twice in two patients, leading to 28 suspected immunologic progressions). Six patients had at least one HIV RNA VL above 2000 copies/mL, and 5 patients had both criteria at the same time for a single blood sample.

However, the progression was only confirmed by a consecutive blood sample in 15 of the 37 patients with suspected progression (i.e. 6.9% of the 217 patients): 10 patients with immunologic progression and 5 with viral progression. Progression could not be confirmed for the 22 remaining patients because either the criteria were not met for the second consecutive sample (n = 15) or ART had been initiated before the confirmatory sample was collected (n = 7).

The median [IQR] follow-up time since enrollment was 37 [35–41] months. At the time of confirmed progression, the median [IQR] HIV RNA VL was 132 [39–858] copies/mL in immunologic progressors and 2210 [2119–3085] copies/mL in virologic progressors, and the median [IQR] CD4+ T cell count was 320 [301–336] in immunologic progressors and 725 [530–759]/mm^3^ in virologic progressors. Representative data for immunologic progression and viral progression are shown in the S1 Fig.

### Analysis of clinical events prior to the immunologic/virologic progressions in HICs

To identify factors that might lead to disease progression during the follow-up of HICs, we analyzed potentially remarkable clinical events in the 3 months preceding the progression. Events were identified in 7 patients. Two of the 5 patients (40%) with virologic progression reported unprotected sexual intercourses in the previous 3 months. However, this potential risk factor was also reported by 63 of the 197 non-progressors (31.9%) having provided information (including patients in stable relationships). The intergroup difference was not statistically significant.

Three patients displayed immunologic progression in the months following an infectious event (diarrhea and *Chlamydia trachomatis* infection in one patient, two episodes of bronchitis in a second patient, and the third patient experienced two episodes of prostatitis, an episode of gastro-enteritis and a whitlow). One patient underwent an epidural injection of corticosteroids in the month before immunologic progression. Lastly, one patient was diagnosed with a B-cell lymphoma four months after immunologic progression. It is noteworthy that apart from the latter patient, neither AIDS nor non-AIDS-related serious adverse events (such as cardiovascular events or cancer) were reported by patients with disease progression, either before or after the progression.

Next, we analyzed clinical and immunologic factors at enrollment in progressors and non-progressors (Table 1). There were no intergroup differences in terms of gender ratio, HLA B57 status or HCV co-infection status. Interestingly, virologic progressors had a shorter median duration of follow-up since HIV diagnosis and were younger than non-progressor HICs. Of note, there were also no differences in terms of nicotine use, systolic blood pressure elevation or body mass index distribution between the three groups. When compared with non-progressors, immunologic progressors had a lower CD4+ T cell nadir (median [IQR]: 496 [376–657.5] vs. 245.5 [220.3–259.8], respectively, *p*<0.001) and a lower CD4+ T cell count at inclusion (median [IQR]: 763.5 [559.3–950.3] vs. 416.5 [296–435], respectively, *p*<0.001).

**Table 1:** Characteristics of the study population at enrollment into the cohort.

	HICs with immune progression (n = 10)	HICs with virologic progression (n = 5)	Non-progressor HICs (n = 202)
Male gender, n (%)	3 (30)	3 (60)	98 (48,5)
Age (years)	48 [43–56]	34 [32–34]***	45 [39–50]
Duration of known HIV infection (years)	18 [13–23]	5 [5–8] ***	13 [8–20]
HLA B57+ (%)	3/9 (33)	1/5 (20)	65/165 (39,4)
HCV+ status, n (%)	3 (30)	1 (20)	44 (22,8)
CD4+ T cell nadir (/mm^3^)	245.5 [220.3–259.8] ***	433 [405.8–455.8]	496 [376–657.5)
CD4+ T cell count (/mm^3^)	416.5 [296–435] ***	643 [527–1447]	763.5 [559.3–950.3]
Ultrasensitive HIV RNA (copies/mL)	117 [12–274]**	118 [78–1023]**	34 [11–89]
Total HIV DNA (copies/10^6^ PBMCs)	11 [11–21]	42.5 [31.5–66.3]*	11 [10–46]
% of detectable VLs during history	35 [17–52] *	32 [17–47]	21 [18–25]

Results are quoted as the median [IQR] or as a percentage. All comparisons were performed relative to the group of non-progressor HICs.

*: p<0.05

**: p<0.01

***: p<0.001

Furthermore, the ultrasensitive HIV RNA VL at enrollment and the frequency of blips prior to the enrollment in the CODEX cohort were higher in immunologic progressors than in non-progressors (Table 1). The ultrasensitive HIV RNA VL at enrollment and the total HIV DNA were higher in virologic progressors than in non-progressors (HIV DNA: 42.5 [31.5–66.3] copies/10^6^ PBMCs vs. 11 [10–46] copies/10^6^ PBMCs, respectively; *p*<0.05).

### Analysis of immune activation parameters prior to the immunologic/virologic progressions in HICs

**Fig 2:**
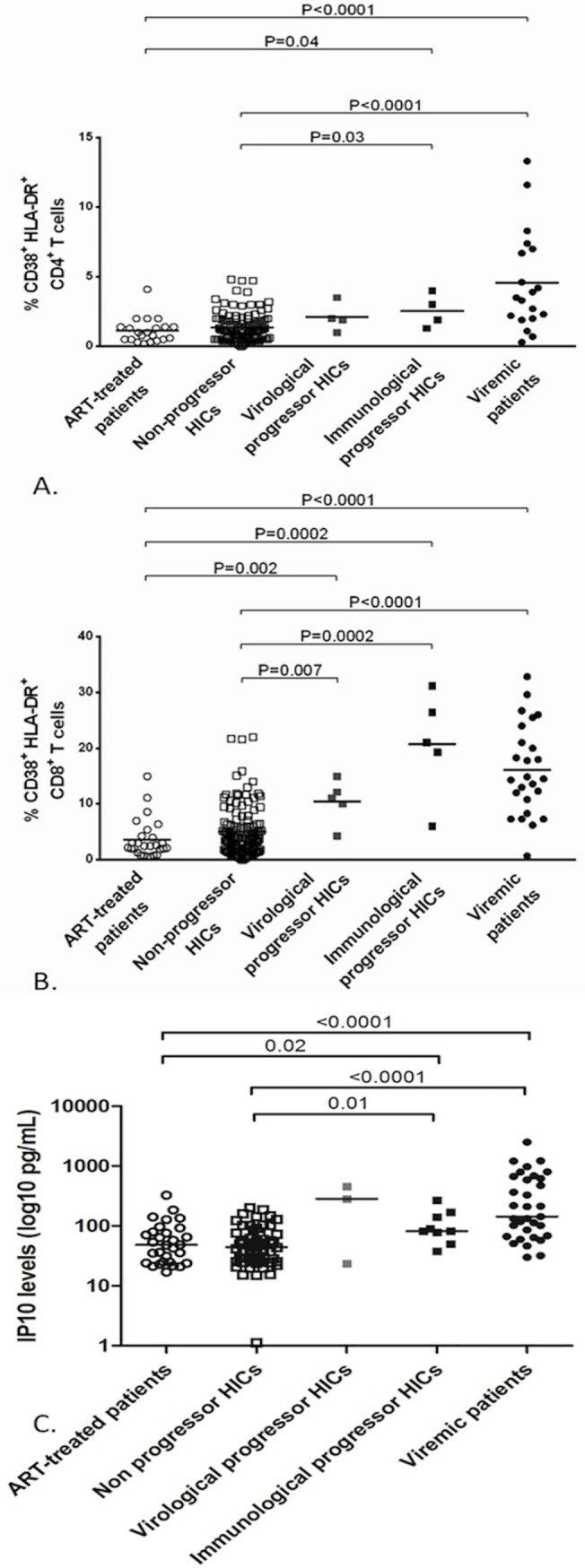
Comparison of immune activation parameters in immunologic or virologic progressor HICs, non-progressor HICs, ART-treated patients, chronic viremic patients and healthy donors. (A) Proportion of activated circulating HLA-DR+CR38+ CD4+ T cells. (B) Proportion of activated circulating HLA-DR+CD38+ CD8+ T cells. (C) Plasma IP10 levels (pg/mL, logarithmic scale).

Lastly, we determined whether or not markers of immune activation/inflammation at inclusion were predictive of progression. As shown in Fig 2, the proportion of activated circulating CD8+ and CD4+ T cells (as defined by the expression of both HLA-DR and CD38 surface antigens) were higher in immunologic progressors than in non-progressors at inclusion in the cohort. In patients with virologic progression, only the proportion of activated CD8+ T cells was significantly higher. Interestingly, the proportion of activated T cells was (i) as elevated in progressor HICs as in viremic patients, and (ii) as low in non-progressor HICs as in ART-treated patients (Fig 2).

Similarly, plasma IP10 levels at enrollment were significantly elevated in immunologic progressors and in viremic patients but were lower in both non-progressor HICs and in ART-treated patients. None other of the biomarkers tested (MCP1, TNFα, IL6, sCD14, sCD163, CRP, and IL10) distinguished between progressors and non-progressors (data not shown).

The various patient groups did not differ in terms of their CD8+ T cells' ability to control HIV replication *in vitro* (data not shown). The ex vivo capacity of HIV-specific CD8+ T cells to suppress HIV-1 infection was similar in the ImmP, the VirP and the non-progressor HICs groups (median [IQR] log p24 decrease: 1.38 [0.52–2.80], 1.65 [0.39–2.99] and 1.18 [0.39–3.06], respectively). Similarly for the ELIspot IFN-γ assays, the distribution was similar among the three groups.

### cART introduction and evolution of progressor HICs

cART was initiated in 11 of the 15 patients with confirmed progression. Interestingly, cART was introduced in 10/10 patients with immunologic progression and only in 1/5 patients with virologic progression. After a median [IQR] follow up of 19 [12.7–25.1] months on cART, the CD4+ T cell count was 389/mm^3^ [296–441]. The HIV-RNA VL was undetectable for all tested patients after 6 and 12 months.

## Discussion

In the present study, we characterized 15 HICs who experienced HIV disease progression with immunologic progression (CD4+ T cell count decrease) or uncontrolled HIV replication from one of the largest, best-defined cohorts of HICs to date (n = 217). Our main conclusions are that (i) a history of profound CD4+ T cell nadir or the presence of long-term, low-level but detectable viral replication (between 50 and 400 RNA copies/ml) and a higher HIV DNA load in blood are major determinants for further disease progression, and (ii) HICs who experience disease progression have higher levels of immunologic activation and inflammation prior to progression. Taken as a whole, our findings highlighted a subgroup of HICs that should benefit from closer follow-up and/or earlier treatment.

When considering only confirmed progressors, the frequency of progression in our cohort was 6.9%. Relatively few data on this topic are available [14,18,20,21]. Okulicz *et al* studied 3 of 25 well-defined elite controllers (12%) having received cART during follow-up [20]. Hunt *et al*. found that 3 (10%) of 30 HICs had CD4+ T cell count below 350/mm^3^ at study entry [14]. These values are similar to the frequency of loss of viral control reported in the largest study to date on the long-term follow-up of HICs (the CASCADE collaboration) [18]. Indeed, Madec *et al* reported loss of virologic control in 22 of 140 HICs (15.7%), after a 20-year follow-up period [18].

Our analysis of purportedly clinically relevant events showed that 2 of the 5 HICs with loss of virologic control had had unprotected sexual intercourse. A similar event may well account for the progression in the patient with *Chlamydia* (a sexually transmitted infection). These events are suggestive of risk of superinfection in these 3 patients. Lack of available plasma prevented us from testing this hypothesis in a phylogenetic analysis [4]. However, superinfections have already been documented in some HICs [25,31], with either maintenance of HIV control [4] or virologic progression [25]. This emphasizes that elite control can be disrupted over time particularly when the immune system is overwhelmed by high viral input. In patients at high risk of superinfection, cART introduction has to be suggested. With respect to immunologic progression, two cases with recurrent bacterial infections and the patient diagnosed with B-cell lymphoma at around the time of progression deserve special attention. These affections might contribute to excessive inflammation with alteration of the CD4+ T cell homeostasis. However, we are not able to draw robust conclusions as to the causality of these factors. In the future, prospective studies must collect exhaustive data at the time of virologic and/or immunologic progression.

In addition to documented occasional events, one must also consider each patient’s history of HIV control. We showed that the CD4+ T cell count at enrollment, the CD4+ T cell nadir, a history of blips, and a higher ultrasensitive HIV RNA VL at inclusion are possible determinants for immunologic progression. We and other previously showed that the presence of blips or a low-level viral replication in HICs is associated with a higher probability of CD4+ cell loss [11]. Changes in CD4+ T cell homeostasis levels and the level of viral replication both appear to contribute to CD4+ T cell attrition and immunologic progression.

Interestingly, the blood level of HIV DNA (which reflects the HIV reservoir) and a higher ultrasensitive HIV RNA VL at inclusion appear to be associated with virologic progression. Also, virologic progressors had a shorter median duration of follow-up and were younger than non-progressor HICs. This latter observation suggests that a longer duration of control without blips could be associated with less exposure to the risk of virologic progression, in line with the notion of “pre-escape” blips reported by the CASCADE consortium [18].

For the first time, we were able to analyze the level of immune activation and systemic inflammation in HICs prior to progression. The literature data suggest that CD4+ and CD8+ activation is positively correlated with the concomitant low-level viremia and negatively correlated with the CD4+ count [14,32]. Here, we showed that patients with higher CD4+ or CD8+ T cell activation and IP10 levels at inclusion are at risk of progression. The higher ultrasensitive HIV RNA VL in immunological and virological progressors can not completely account for this association. Indeed, T cell activation and IP10 levels are as elevated in progressor HICs as they are in treatment-naïve viremic patients contrasting with strikingly different levels of plasmatic VLs. We suggest that these parameters should be taken into account when planning follow-up and care for HICs.

Although cART is now recommended for all HIV-infected patients, the systematic treatment of HICs is subject to debate [33]. Interestingly, our present results and some literature reports [14,20] show that most HICs initiating cART presented with immunologic progression alone. An analysis of the effect of cART on the change over time in the CD4+ count was beyond the scope of the present study. However, some researchers have evaluated the timing strategy for cART in HICs [12,34,35]. While cART always has an effect on the HIV VL, the efficacy for CD4+ T cell reconstitution is variable. In fact, HICs display slow CD4+ T cell recovery during cART [12]—suggesting that earlier initiation of therapy could be beneficial in HICs with immune activation and a falling CD4+ T cell count.

In conclusion, HICs constitute a heterogeneous group of HIV-infected patients. As recently pointed out, the definition of HIC varies [36,37]. Our results show that about 7% of a French cohort of HICs experienced virologic or immunologic progression over a 5-year period. The history of HIV infection (CD4+ T cell nadir and kinetics, low-level HIV RNA VL, blips, and the HIV DNA load) and levels of baseline immune activation and inflammation are major determinants of progression. All these parameters should be taken into account when stratifying at-risk patients, in order to adjust their follow-up and optimize the time at which cART is initiated.

## Supporting Information

S1 Fig Examples of immunologic progression (Fig A) and virologic progression (Fig B). (TIF)
