# Supplementary figures and images for: Immunologic and Virologic Progression in HIV Controllers: The Role of Viral “Blips” and Immune Activation in the ANRS CO21 CODEX Study
Source: PLoS One. 2015 Jul 6;10(7):e0131922. doi: 10.1371/journal.pone.0131922 (PMC4493076; doi:10.1371/journal.pone.0131922)

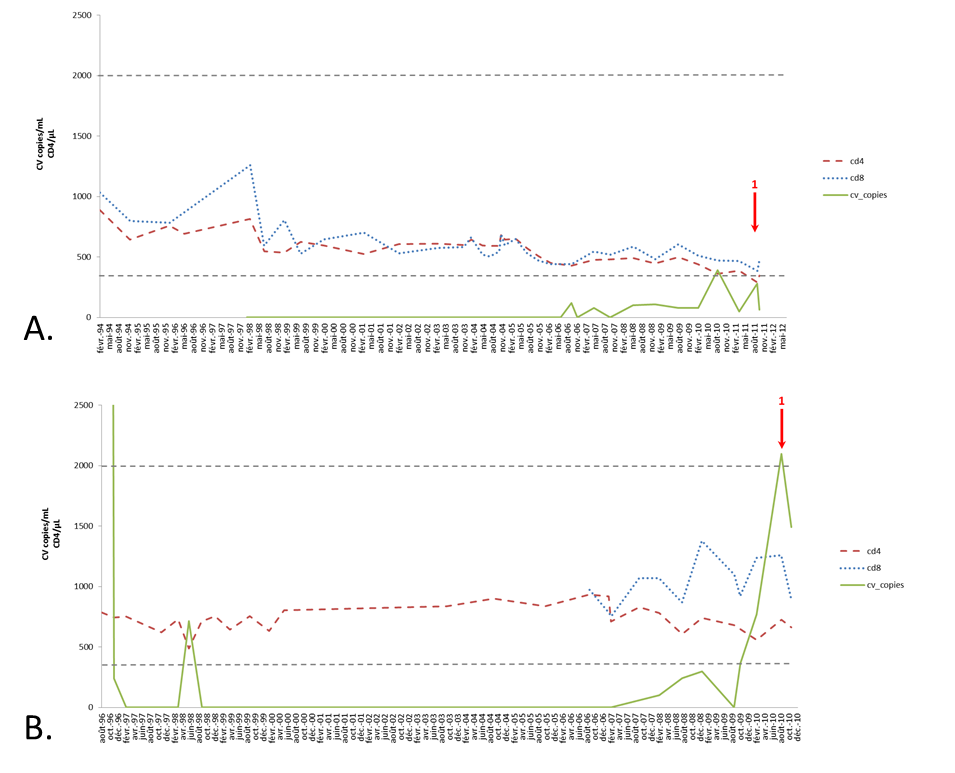

Supplement: S1 Fig — (TIF) [file pone.0131922.s001.tif]
